# Impacts of Human Activities on Ecosystem Service Value in Arid and Semi-Arid Ecological Regions of China

**DOI:** 10.3390/ijerph182111121

**Published:** 2021-10-22

**Authors:** Xin Fan, Haoran Yu, Damien Sinonmatohou Tiando, Yuejing Rong, Wenxu Luo, Chan Eme, Shengya Ou, Jiangfeng Li, Zhe Liang

**Affiliations:** 1 School of Public Administration, China University of Geosciences Wuhan, Wuhan 430074, China; worldwin2020@sina.com (X.F.); damientiando90@gmail.com (D.S.T.); emechan340@gmail.com (C.E.); 2 State Key Laboratory of Earth Surface Processes and Resource Ecology, Beijing Normal University, Beijing 100875, China; 3 Anhui Urbanization Development Research Center, Hefei 230022, China; watt2020521@163.com (H.Y.); liazhe_320@163.com (Z.L.); 4 State Key Laboratory of Urban and Regional Ecology, Research Center for Eco-Environmental Sciences, Chinese Academy of Sciences, Beijing 100085, China; yjrong_st@rcees.ac.cn; 5 University of Chinese Academy of Sciences, Beijing 100049, China; 6 International Education College, China University of Geosciences, Wuhan 430074, China ; intlstudents@cug.edu.cn; 7 School of Geography and Tourism, Shaanxi Normal University, Xi’an 710119, China; osy201705598@snnu.edu.cn

**Keywords:** ecosystem services value, human activities, dynamic mechanism, arid and semi-arid regions, special regression

## Abstract

The quantitative and spatial–temporal variations in the characteristics of ecosystem value can be helpful to improve environmental protection and climate adaptation measures and adjust the balance between economic development and the ecological environment. The arid and semi-arid regions of China are undergoing the effects of climate change across the entire northern hemisphere. Their ecological environments are fragile and in conflict with anthropogenic activities, which significantly altered more ecosystems services in these regions. Therefore, estimating the effects of anthropogenic activities on ecosystem services is important for formulating ecological policy and regional environmental mitigation plans of these regions. This study employed the model of ecosystem service value (ESV) assessment and the bivariate spatial autocorrelation method to reveal the spatiotemporal variations in the characteristics of ecosystem value in the arid and semi-arid ecological regions of China and its interaction with human activities. Results showed that (1) the total value of ES of the study area increased from USD 487,807 billion in 2000 to USD 67,831,150 billion 2020; (2) the ES value provided by forest land first increased by 5.60% from 2000 to 2020; (3) the ESV provided by grassland showed an overall decline over the 20 years. Food and raw material production showed the lowest ES value, and climate regulation and soil conservation decreased from 2000 to 2020; (4) the index of human footprint patches decreased from 45.80% in 2000 to 17.63% in 2020, while the high and very high human footprint index areas increased significantly, mainly due to the rapid urbanization and improvement of railway networks in these areas. Spatially, the regions with high human footprint were mostly dispersed in the northeastern of China such as Shanxi and Gansu, whereas the regions with a low human footprint remained mainly located in the central and southwestern parts of China; (5) significant spatial dependencies between changes in ESV and the human footprint index were recorded. Our study could provide a scientific basis for ecosystem functions regulation and land development security in arid and semi-arid ecological regions.

## 1. Introduction

The continuous degradation of ecosystem functions is a common challenge facing humankind, and it is the focus of global sustainable development research. The arid and semi-arid regions account for approximately 40% of the world’s land area. However, the intensification of human activities has caused a series of environmental problems exerting greater pressure on ecosystems services, land desertification and reduced vegetation, and increased pollution [1]. The loss of the natural environment and agricultural land are particularly severe, and thus, the sustainable development of the local society and economy has been greatly affected [2,3].

Ecosystem services denote the various benefits that humans obtain from the ecosystem, as well as supply services, regulation services, support services, and cultural services [4]. Nevertheless, vigorous economic development activities have had a huge impact on ecosystems replicated directly or indirectly and degradation of terrestrial ecosystem services [5]. Principally in arid and semi-arid areas, the vegetation provides vital goods and services for the local ecosystem, due to battling the desertification process in those regions [6]. The Millennium Ecosystem Assessment warned that 60% of the ecosystems that humans depend on are in a state of continuous degradation across the world [7]. Global land use change induced by human activities has been among the driving forces behind changes in ESV. The arid and semi-arid regions of China are the “sensitive areas” of climate change in Asia and even the entire northern hemisphere, and the ecological environment is fragile. However, these areas have undergone a series of ecological maintenance projects over the past 20 years, and their ecosystems have been improved to some extent [8,9]. Currently, the pressure of human activities on ecosystems services changes in these areas still continues. Therefore, the quantitative description of its temporal and spatial evolution characteristics can provide an important reference for formulating reasonable ecological protection policies and improve the current ecological environment protection.

After the premise of the scientific evaluation of global ecosystem services value by Costanza et al. [10], the Chinese scholar Xie Gaodi [11] conducted a questionnaire survey in 2002 among 200 professionals with ecological backgrounds, modified the coefficient according to the actual situation of China, and obtained the land ESV with China as the scale. Gaodi established a table of “Ecosystem Service Value Corresponding per Unit Area of China’s Ecosystem” that fits China’s national conditions, providing an important basis for the calculation of ES value in China. Research on the concepts, values, and trade-offs involved in ecosystem services has become a hot issue in related disciplines at home and abroad and has gradually been applied to regional land use planning and decision-making. For example, Zhang et al. [12] have revealed a correlation between the temporal and spatial change in the characteristics of Xinjiang’s ecosystem service value and the impacts of human activities in ecological arid regions. Menzel et al. [13] analyzed the relationship between ecosystem trade-offs and synergy in Monterey Bay, California, USA. Thapa and Murayama et al. [14] took Hanoi, Vietnam, as an example, and explored the impact of human activity on soil and water resources in the arid area of Hanoi, Vietnam. The spatial relationship between ecosystem services and urbanization in the middle reaches of the Yangtze River from 1995 to 2015 has been analyzed by Wanxu using the logistic regression model [15]. Due to the characteristic sensitivity of these regions to global changes and the complexity of human pressures, the research still needs to be improved. The fragility of the ecosystems in these areas is determined by their inherently harsh physical conditions including sparse vegetation, continental climate, sandy soils, and water shortage, which make them susceptible to desertification [16,17]. The increase in the scope of human-caused activities has greatly affected the structure of the ecosystems [18,19]. It is valued that 35% of Inner Mongolia’s remaining grassland area is classified as degraded, and 22% is seriously degraded; in Xinjiang, 75% of the grasslands are degraded [20]. Land cover degradation in northern China had been accelerating in the last five decades, with annual expanded rates of 1560 km^2^ between the late 1950 s and 1975, 2100 km^2^ between 1975 and 1987, and 3600 km^2^ from 1987 to 2000. Desertification has a significant impact on the climate in China, with droughts becoming increasingly frequent in arid and semi-arid regions [21,22]. Few studies have been carried on ecosystem services valuation over the entirety of China’s arid and semi-arid areas ecological regions, and these are deemed to be areas that are sensitive to climate change across the entire northern hemisphere. Therefore, altering the temporal and spatial evolution characteristic of ES value in these regions is of great significance for the formulation of future policies regarding climate change. The objective of this study is (1) examine the spatial–temporal variation in ES value of China’s arid and semi-arid ecological regions for a time period of 2000–2020 and (2) to identify the importance of human footprint in impacting the changes in ES value in the study area throughout time and space. 

## 2. Outline of Study Area and Data Foundation

### 2.1. Outline of Study Area

**Figure 1:**
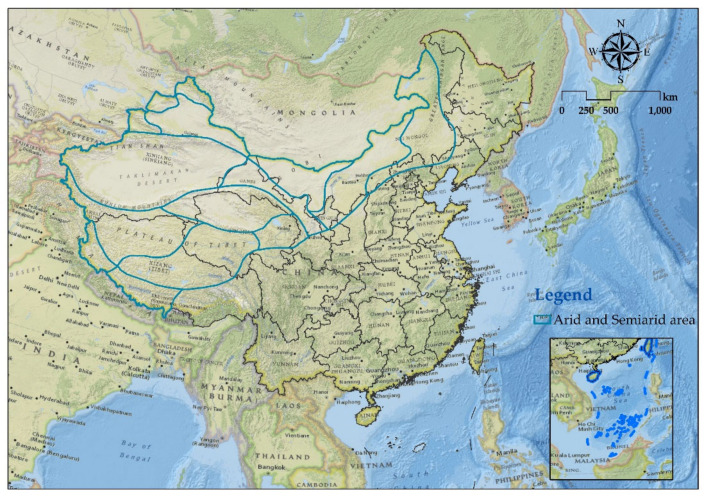
The physical location of the study area.

China’s arid and semi-arid ecological regions comprise Xinjiang Uygur Autonomous Region, Tibet Autonomous Region, Qinghai Province, Gansu Province, Ningxia Hui Autonomous Region, Inner Mongolia Autonomous Region, Shaanxi Province, Shanxi Province, Hebei Province, Liaoning Province, etc. (Figure 1), for a total area of 4,536,870.60 km^2^, accountancy for 47.3% of China’s total land area [23]. Due to the uneven distribution of precipitation within and between years in the study area, droughts still occur frequently, soil erosion by wind is very serious, and farmland being buried by quicksand is a common phenomenon. Since the reform and opening-up policies, a large number of forested areas have been harvested, grassland has been overloaded, grazing has occurred, wasteland has been put into use, and the planting of food has destroyed the ecological environment of the study area [24,25]. However, during the last 20 years, the implementation of projects addressing ecological protection, such as wetland restoration, ecological water transport, and tree planting (grass), encouraged humans to move to this area. The activities and ecological environment have undergone major changes. The temporal and spatial changes in the ecological environment need to be explored, and the overall economy of the study area needs to be further developed.

### 2.2. Data Foundation and Processing

We used the satellite imagery data from 2000, 2010, and 2020, obtained from Resources and Environmental Sciences, Chinese Academy of Sciences (https://www.resdc.cn/ accessed on 20 October 2018). These land-use datasets with 30 m resolution were developed in 2000, 2010, 2020, respectively, included land categories into cultivated land, construction land, forest land, grassland, water bodies, wetland, deserts, and other 7 types of land use (Table 1). The accuracy of the 7 classes of land use was above 94.3% [26]. Population density and GDP data were from the three years of 2000, 2010, and 2020 and were sourced from the Resource and Environment Science Data Cloud Platform of the Chinese Academy of Sciences (https://www.resdc.cn/ accessed on 20 October 2020). The night light data with a spatial resolution of 500 m came from the three years of 2000, 2010, and 2018. Due to the lack of data for 2020, the 2018-night light data were used as a substitute. The data came from the Harvard Dataset’s platform (https://dataverse.harvard.edu/ accessed on 20 October 2020) and were processed by Chen Zuoqi et al. [27]. All data were sampled to a uniform resolution of 1000 m.

## 3. Research Methods

### 3.1. Measurement Ecosystem Service Value

In order to eliminate the influence of factors such as grain fluctuations and currency inflation on the estimation results, we calculated uniformly using the average price of grain in China in 2007 as the benchmark price [28,29]. Grain prices are the average of grain prices in each province in China. According to the statistical yearbook, the average of grain prices in all regions of China that year should include all rural areas. We found that the economic value of the 1-unit equivalent factor in the study area is USD 1231.90 billion n/hm^2^∙year and the ecological system service unit price. Since land-use types cannot correspond to ecosystem types one to one, in this study, we selected the closest land-use type for equivalent assignment. In this study, construction land is considered to not provide ecosystem services. The value equivalent of ecosystem services per unit area for China’s ecosystem in 2007 is shown in Table 2. The calculation formula and introduction refer to the related research [30,31].

In fact, due to the spatial heterogeneity of the ecological service value, even the same ecosystems have different internal ecological service values. Existing research results show that the ecological service value is proportional to its biomass. Therefore, the above-mentioned land ecological service value can be revised according to the difference in the biomass of various regions. The formula is as follows:

(1) $$E_{j}^{∲}=\frac{b_{j}}{B}\cdot E_{1}$$

In Formula (3), $E_{j}^{‘}$ is the revised ecological service value of the biomass; $E_{1}$ is the ecological service value before it is revised;$\;b_{j}$ is the biomass of the category  j evaluation unit; B is the average biomass of the study area. It is difficult to calculate biomass directly, and various studies have shown [23,32] that biomass and vegetation coverage is directly proportional and that vegetation coverage can be calculated from the normalized vegetation index (NDVI) using the corresponding relationship. 

Normalized difference vegetation index (NDVI) is used to qualitatively and quantitatively evaluate vegetation coverage and its growth vitality. We can also simply understand it as an indicator reflecting vegetation density and health. The NDVI value of the evaluation unit can be summarized and the ecological service value revised using the following formulae:

(2) $$f=\frac{NDVI-{\mathrm{NDVI}}_{min}}{NDVI_{max}-{\mathrm{NDVI}}_{min}},$$

(3) $$F_{i}=\frac{f_{i}}{\bar{f}},$$

(4) $$E=E_{i}\times F_{i},$$

where f is the vegetation coverage, $F_{i}$ is the vegetation coverage revision coefficient of the *i*-*th* grid, $f_{i}$ is the vegetation coverage of the i−thi−th grid, $\bar{f}\bar{f}$ is the average vegetation coverage of the study area, $E_{i}\;$ is the revision of the ecological service value before, and E is the revised ecological service value. Thus, the revised value of the ecological service value of each grid can be obtained (Table 3).

$NDVI_{max}$ represents the highest vegetation coverage value in the study area, indicating that there is more woodland and grassland in this area, and the overall ecosystem service function will be relatively high. ${\mathrm{NDVI}}_{min}$ represents the lowest vegetation coverage value in the study area, and ${\mathrm{NDVI}}_{min}$ represents the current for the vegetation coverage of grid species; the grid range of this study is 20 km, so the NDVI value of each grid is the mean value within the grid. Using the above formula, the adjustment coefficient is based on the vegetation coverage index as a correction, which can more subtly reflect the overall spatial difference between the ecosystem service values. The vegetation is quantified by measuring the difference between near-infrared (vegetation strong reflection) and red light (vegetation absorption). The value range is (−1,1). If the value is close to −1, it means this area is possibly a body of water. If the value is close to 1, it is likely to be dense green leaves; NDVI = (NIR-Red)/(NIR + Red). Among them, NIR refers to the reflectivity in the near-infrared band, and Red refers to the reflectivity in the red band. The NDVI value is large, and the ecosystem service value is relatively high. In order to more closely reflect the spatial difference of ecosystem service value, in the cell-scale ecological service value revision, the vegetation coverage coefficient was selected as an indicator, and the cell-by-cell ecosystem service was performed according to the corresponding relationship between vegetation coverage and NDVI value revision. 

### 3.2. Human Footprint Index

Sanderson et al. proposed for the first time to characterize human activities based on the human footprint index (HFI) and evaluate the impact of human activities on nature on a global scale [33,34,35,36]. Based on the human footprint index method and according to the development characteristics of arid and semi-arid ecological regions in China and the availability of data, the intensity of human activities, population density, night light index, and per capita GDP were finally selected to represent the degree of human activity. We standardized all data to values between 0 and 1, performed weighted summation, and used ArcGIS to process the human footprint index for the years 2000, 2010, and 2020. The human activity intensity index model used to describe the overall impact of human activities on the ecosystem service value in the study area calculation formula is

(5) $$HAI=\frac{\sum_{i=1}^{N}A_{i}P_{i}}{TA},HFI=HAI+POP+GDP+NTL$$

where HFI is the human footprint index, POP is the population density, GDP is the economic development status, NTL is the night light index, HAI is the human activity intensity index, N is the number of land-use types, $A_{i}$ is the land-use type area, $P_{i}$ is the human activity intensity coefficient reflected by the ecological value, and TA is the total area. In this paper, the Lohani inventory method, the Leopold matrix method, and the Delphi method were used to determine the human activity intensity coefficient.$P_{i}$ is the average value of the person was taken as $P_{i}$, which was substituted into the human activity intensity index evaluation model for calculation (Table 4).

### 3.3. Dynamic Mechanism between Human Footprint and Ecosystem Services Value

Due to the spatial autocorrelation of the classic linear regression model, when solving problems, there will be an incorrect estimation of the model parameters and a reduction in the effectiveness of the model. Therefore, it is necessary to build a regression model suitable for spatial data. Anselin gave the general form of the spatial regression model [37,38,39] as follows:

(6) $$Y=\rho W_{1}+X\beta +u,$$

(7) $$\mu =\lambda W_{2}\varepsilon +\mu ,\mu \sim N\left[0,\sigma^{2}I\right]$$

The following models can be derived from the general form of the spatial autoregressive model. When ρ and β are not equal to 0 and λ is equal to 0, it is a spatial lag model (SLM). In this model, the dependent variable of the studied region is related to the explanatory variable in this region and to the dependent variable in the adjacent region. When ρ is equal to 0 and β and λ are not equal to 0, it is a spatial error model (SEM)—that is, the dependent variable YY of the studied region is related to the explanatory variable X of this region as well as to the dependent variable and the adjacent region. The explanatory variable is also related. Spatial dependence refers to the lack of independence of observations in space but also to the data structure underlying this spatial correlation—that is to say, the intensity and pattern of spatial correlation have the same absolute position (pattern) and relative position (distance). This article used SEM and SLM to explore the mechanism of interaction between human activities and ecosystem service values.

## 4. Results Analysis

### 4.1. Spatiotemporal Patterns of Ecosystem Service Value

Temporally, the ES value provided by ecosystems in China’s arid and semi-arid ecological regions followed an overall upward trend from 2000 to 2020, rising from USD 487,807 billion in 2000 to USD 67,831,150 billion in 2020 (Table 5), indicating that between 2000 and 2010 there was an increase of 3.45%, and from 2010 to 2020, there was an increase of 1.06%. This is related to the intensive human activities carried out in this area in the past 20 years, such as a succession of ecological projects, including tree planting in the desert, and grass planting, which have increased the ecosystem services of the study area. During the study period, the value of ES provided by forest land first increased and then decreased, increasing by 5.60% from 2000 to 2020 (Table 6). The value of ecosystem services providing by grassland first decreased and then increased but showed an overall decline over the 20 years. The value of system services continued to increase from 2000 to 2020; the ES value provided by the desert system showed the lowest contribution. The arid and semi-arid regions of China provide a relatively high proportion of soil function, hydrological regulation, and maintenance of biodiversity in the ecosystem structure of China’s arid and semi-arid regions. The value of services provided for food and raw material production is low. It is worth noting that the overall trend of climate regulation and soil conservation decreased from 2000 to 2020.

**Figure 2:**
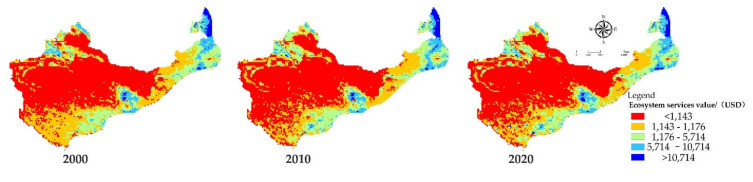
Spatial distribution of ecosystem service value at 20 km scale in 2000, 2010, and 2020.

Spatially, change and differentiation in the ES value of the study area, seen in Figure 2, shows that the regions with high-value areas of ecosystem services were mostly concentrated in the Greater Khingan Mountains, the Yellow River Basin, and the Tarim Basin, while the regions with low-value areas were mainly located in arid areas such as the Alxa Plateau, the Taklimakan Desert, and the high slopes of the loess. In addition, the main prefecture-level cities and surrounding grids were also areas where the ES value reduced significantly, mainly because these areas had a relatively high level of urbanization, while at the same time, they have a strong impact on the ecosystem.

### 4.2. Spatiotemporal Patterns of Human Footprint in Arid and Semi-Arid Regions of China

**Figure 3:**
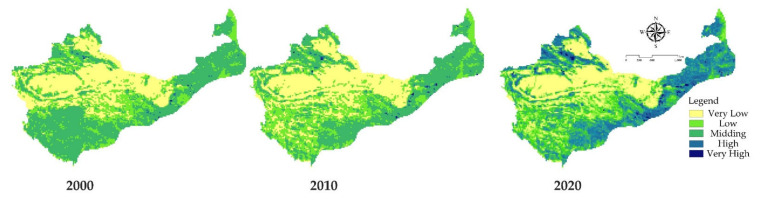
Spatial distribution characteristics of the average human activity intensity on a 20 km scale in 2000, 2010, and 2020.

In ArcGIS 10.6, the natural breakpoint method was used to divide the results of the human footprint index into five levels—namely, very low (0–0.2), low (0.2–04), medium (0.4–0.6), high (0.6–0.8), and very high (0.8–1.0). The human footprint classification and the temporal and spatial distribution pattern of human activities in the study area were obtained (Figure 3).

In terms of time and scale, the human footprint index of China’s arid and semi-arid regions changed significantly from 2000 to 2020. Among them, the areas with a high human footprint and very high human footprint continued to expand, which is very important for China’s western development strategy. With the acceleration of urbanization in development activities, the areas of human activities have become more and more extensive. It is worth noting that the low human footprint areas were mainly located in the central and southwestern regions of the study area during the 20 years investigated. The high human footprint areas are mainly distributed in the three northeastern provinces close to the central and northeastern regions of China, as well as areas such as Shanxi and Gansu. Due to the large population, in the past 20 years, with the rapid development of cities and towns, and the improved transportation accessibility, the scope of human activities in these areas has increased significantly.

From a spatial scale, the areas with large human footprints in the arid and semi-arid regions of China from 2000 to 2020 were mainly distributed in the southeastern edge of the study area, such as Hebei, Shanxi Yanbei, northern Shaanxi, Xihaigu in southern Ningxia, Dingxi, Gansu, and Yuzhong. It can be seen from Table 7 that the number of medium human footprint index patches decreased from 45.80% in 2000 to 17.63% in 2020, while the high and very high human footprint index areas increased significantly, mainly due to the rapid urbanization and improvement of railway networks in these areas. The rapid development of the highway networks has led to the expansion of the human footprint in these areas.

### 4.3. Impact of Human Activities on Ecosystem Services Value in Arid and Semi-Arid Regions of China

Based on the GeoDa 1.18 platform, we measured the bivariate global autocorrelation Moran’s I index between the value of ecosystem services and the intensity of human activities at the 20 km grid scale in 2000, 2010, and 2020. In 2000, 2010, and 2020, the bivariate global autocorrelation Moran’s I index between the value of ecosystem services and the intensity of human activities at the 20 km grid scale were 0.412, 0.461, and 0.495, respectively, and they were significant at the level of 0.001. The bivariate spatial autocorrelation test showed that there was a significant spatial dependence effect between the ecosystem service value and human footprint, and a significant positive externality was generated during the research period (Table 8). Different from the results obtained in other research regions in China, the value of ecosystem services in China’s arid and semi-arid regions gradually increased. Compared with other regions in China, such as coastal areas, Anhui Province, Henan Province, and other central regions, China’s arid and semi-arid regions have relatively poor endowments, and deserts account for a larger proportion of land-use types, accounting for 41.23% of the study area in 2000. However, forest land and wetland, which can provide a higher ecosystem service value, account for lower proportions, at 3.76% and 1.37%, respectively. After the implementation of the western development strategy in 2000, the overall development strategy has been to carry out infrastructure development and economic development while protecting the ecological environment. The area of human activities has increased over the 20-year period, but the ecosystem service value of China’s arid and semi-arid regions has not. Therefore, the decrease is mainly due to the increase in the proportion of forest land and wetland, which can provide a higher ecosystem service value. By 2020, the amounts of the two types of land will be 4.23% and 2.03%, respectively (Table 9 and Table 10).

**Figure 4:**
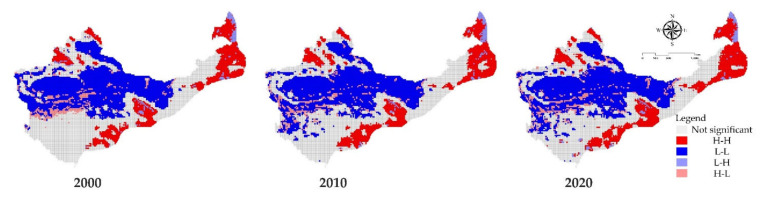
LISA cluster map of human footprint index and ecosystem service value space in the study area on a 20 km scale in 2000, 2010, and 2020.

The LISA cluster map shows the bivariate local autocorrelation between the ecosystem service value and the intensity of human activities at the 20 km grid scale in 2000, 2010, and 2020 (Figure 4). Comparing the bivariate local spatial autocorrelation LISA cluster map between the ecosystem service value and human footprint level at different time points can allow us to find similar spatial characteristics. During the study period from 2000 to 2020, the HH type (highland average ecosystem service value and high human footprint level type area) and LL type (low land average ecosystem service value and low human footprint level type area) were the main clusters types of the study area. It is worth noting that in the urban areas of the study area, the LH type (low land average ecosystem service value and high human footprint type area) appeared. This spatial agglomeration phenomenon was found in many similar research results. In developed urban areas, due to the expansion of construction land, the ecosystem service value decreased across the research unit. In the middle of the study area, the HL type (highland average ecosystem service value and low human footprint type area) appeared. This phenomenon showed a strong correlation to tree planting and afforestation. The levels of wind and sand in northwest China are very high and have serious impacts. To prevent wind and sand fixation, many plants such as Elaeagnus sylvestris have been planted in the arid areas of the northwest to improve the living environment. This has effectively improved the ecological environment of the region and increased the value of ecological services there.

## 5. Discussion

### 5.1. Dynamic Mechanism between Human Activities and Ecosystem Services Value

As far as the drylands regions of China are concerned, the ecosystem services in the semi-arid regions are more affected. The main reason for this situation is that the harsh climate and living environment of the region make it unsuitable for humans, animals, and animals. Plants, etc. can survive. Figure 3 shows that the low-value areas are mainly distributed in regions with more arid climates, while the climate in the northwest and southeast areas of the study area is relatively comfortable and suitable for high levels of human and animal biological activities. Therefore, high–low values (HL), low–high values (LH), and high–high values (HH) are mainly distributed in semi-arid areas. From the spatial regression model, we found that the value of ecosystem services is affected by its grid cell elements but also by neighboring grid cells or more distant grid cell elements. This means that the concentration of population and economy does not automatically lead to the degradation of ecological services [40,41,42].

Between 2000 and 2020, the relationship analysis between human activities and ESV showed that the overall value of regional ecosystem services has an upward trend, exhibiting HH type (highland average ES value and high human footprint level type area) and LL type (lowland average value and low human footprint level type area) trends, which shows that people’s beneficial activities have greatly promoted the ecological improvement of ES value; however, these findings are in contradiction with Chen et al., who showed that the high and low value of ecosystem services are recorded, respectively, in eastern and western regions in Zhejiang, Fujian, and Guangdong. This may be due to the direction of the western region, which is a province with low ecosystem services and is sparsely populated. With the improvement of the original land use plan, and through large-scale afforestation and ecological water transportation, the original ecological environment has been improved, the quality of the ecological environment has been increased, and the value of the ecosystem services per capita has been raised. Therefore, future land use planning and ecological system protection planning processes should comprehensively consider cross-regional coordinated governance, which will improve land use planning and ecological protection.

### 5.2. Comparison of Human Disturbances Impact on Ecosystem Services Value

Figure 2 and Figure 3 show that the areas with high value of ecosystem services and human activities in the study area are mainly concentrated in semi-arid areas, such as Shanxi and Gansu. These areas are mainly located at the junction of a semi-arid and humid climate. Compared with other areas in the study area, in these areas, it is easier for species to survive, and here, it is suitable to carry out large-scale construction activities for humans, leading to the more frequent restoration of the ecological environment and human activities. By comparing our results with previous research on arid and semi-arid areas, it can be found that [29,30,31,32] the main activities that affect the ecosystem are the improvement of the wetland environment, the creation of artificial oases, and ecological water transport, and these improvement activities are mainly conducted in semi-arid areas. The main reason for this is strictly related to the scope of human activities. Compared with other regions, human activities in semi-arid areas are more concerned with the construction of the ecological environment, as the people in these areas live in a harsh living environment. In addition, in the 1970s and 1980s, the country implemented a “grain-based” agricultural development strategy. The total area of reclaimed grassland has been estimated to reach 27 million hectares, accounting for 25% of the country’s total cultivated land at that time, causing great damage to the natural environment [30,32]. Since then, the country has successively promoted and implemented the “Three Norths” shelterbelt system, the grass restoration project, and the soil and water conservation project. These key projects in semi-arid areas have caused an increase in human activities. 

### 5.3. Policy Implications

As far as China’s arid and semi-arid ecological regions are concerned, it is still a top priority to ensure the balance between economic development and ecological environment protection. It is necessary to follow the principle of ensuring the coordination of ecosystem functions, consider social and economic development and ecological function, and establish a district-differentiated utilization management system. For the Yellow River Basin, it is necessary to reduce all kinds of pollution, reduce the pressure of rivers and lakes for receiving sewage, and strengthen the preservation and appreciation of high-value ecosystem services. For arid areas, where the ecological environment is more severe, the vegetation coverage has increased in the past 20 years, and ecological functions have increased significantly [43,44]. Through studies on the Manas River Basin and the Tarim River under ecological restoration measures such as ecological water delivery, the ecological environment was found to have improved significantly [45,46,47]. Therefore, it is necessary to form a set of targeted models and operating mechanisms for ecological restoration policies in different regions. Currently, the transformation of ecological services into ecological products with valuable attributes is an important measure to achieve a benign cycle [48,49], such as the use of water and forests in the natural ecosystem to develop ecological agriculture or carry out ecological farming based on “ecological elements.” The eco-cultural tourism industry, which has aesthetic and cultural value, can achieve the conciliation of economic development and ecological environmental protection by realizing the activation of the ecosystem [50]. Based on operating similar models, the government or society must complete a series of measures to ensure the realization of the value of ecological products, such as the improvement of the natural resource property rights system and the ecological compensation system [51,52].

### 5.4. Validity and Uncertainty of This Study

The commonly used evaluation methods are mainly based on the unit service function price and the unit area value equivalent factor [53,54,55]. The two methods were different in the calculation models and parameter selection, so the evaluation results were also quite different. The use of different standard calculation prices or evaluation methods in the same area will cause the service value of each ecosystem to become too enlarged or reduced. Additionally, the equivalent factor method uses land-use and land-cover data as the data source; this can more intuitively show the impact of anthropological activities on the structure, process, and function of the ecosystem and is convenient for spatial analysis combined with GIS. Therefore, this study adopted this method. However, the equivalent factor method itself has certain shortcomings. For example, the value of the equivalent factor is more dependent on the researcher’s setting of correction methods and parameters, which will cause differences in the evaluation results. In addition, due to the heterogeneity, complexity, and dynamic characteristics of the ecosystem, its service functions also show obvious temporal and spatial differences. The spatial autocorrelation analysis method can be used to intuitively describe the macro-scale characteristics of the spatiotemporal evolution of ecosystem service value and intensity of human activities, but the local abnormal information in the evolution process is difficult to effectively identify. Therefore, a more accurate evaluation model is needed to facilitate the identification of the characteristics of non-linear changes in ecosystem service value and local specific information at different scales.

## 6. Conclusions 

Studying the impacts of human activities on ecosystem value changes in arid and semi-arid ecological regions of China, we found that there are strong dependencies between human activities and changes in ESV. The following conclusions are drawn:

(1) From 2000 to 2020, the total ESV of the study area increased from from USD 9268.30 billion in 2000 to USD 9690.21 billion in 2020 (Table 5), indicating that between 2000 and 2010, there was an increase of 3.45%, and from 2010 to 2020, there was an increase of 1.06%.
(2) The ESV of grassland showed an overall decline tend over 20 years of the study period. The ESV of cultivated land increased during our study period, whereas the ESV provided by the desert system recorded declined.
(3) In terms of human activities, the low human footprint units were located in the central and southwestern regions, whereas the high human footprint units were mainly distributed in the northeastern regions including the provinces of Shanxi and Gansu. Due to the large population and the rapid development of cities and towns the scope of human activities in these areas has increased significantly.
(4) During the study period, significant spatial dependencies between the variation in ESV and the human footprint were recorded. The regions with high human footprint units ESV increased during the study area, which had a significant positive effect at the 0.001 level. The SEM model showed that every 1% increase in the human footprint index in 2000, 2010, and 2020 increased the value of ecological services by 0.047%, 0.080%, and 0.054%.

Outcomes from this study suggest that anthropogenic activities in such a fragile ecological environment must be pursued with attention, which could help policymakers to take efficient measures in light of the local conditions and the global problem of climate change.

## Figures and Tables

**Table 1:** Equivalent land use categories between the raw data.

Land Type	Corresponding Original Data Land Type
Forest	Woodland, shrubland
Grass	High-coverage grassland, medium-coverage grassland, and bottom-coverage grassland
Wetlands	Tidal flats, beaches, marshes
Lakes and Rivers	Canals, lakes, reservoirs, and ponds
Desert	Sandy land, bare land, bare rock texture, other unused lands, permanent glacier, and snow
Farmland	Paddy land, dry land
Construction land	Urban areas, rural residential areas, industrial areas, and transportation construction land

**Table 2:** Value equivalent of ecosystems services per unit area for China’s ecosystem in 2007.

Primary Type	Secondary Type	Forest	Grass	Farmland	Wetlands	Lakes and Rivers	Desert	Construction Land
Provision of services	Food production	0.33	0.43	1.00	0.36	0.53	0.02	0.00
	Raw material production	2.98	0.36	0.39	0.24	0.35	0.04	0.00
Regulation service	Gas regulation	4.32	1.50	0.72	2.41	0.51	0.06	0.00
	Climate regulation	4.07	1.56	0.97	13.55	2.06	0.13	0.00
	Hydrological regulation	4.09	1.52	0.77	13.44	18.77	0.07	0.00
	Waste disposal	1.72	1.32	1.39	14.40	14.85	0.26	0.00
Support service	Conserve the soil	4.02	2.24	1.47	1.99	0.41	0.17	0.00
	Maintain biodiversity	4.51	1.87	1.02	3.69	3.43	0.40	0.00
Cultural service	Provide an aesthetic landscape	2.08	0.87	0.17	4.69	4.44	0.24	0.00
	total	28.12	11.67	7.90	54.77	45.35	1.39	0.00

**Table 3:** China’s arid and semi-arid ecological regions in 2000, 2010, and 2020: correction coefficients and ecosystem service values.

Land-Use Type	Correction Factor			Ecosystem Service Value/(USD∙hm^−2^∙Year^−1^)
	2000	2010	2020	2000–2020
Forest	2.363776	2.107235	2.220137	4948.7
Grass	1.193545	1.249497	1.280196	2053.8
Farmland	1.813535	1.893931	2.010917	9732.0
Wetlands	1.42477	1.39938	1.221102	1390.3
Lakes and Rivers	0.828647	0.833241	1.145649	7980.8
Desert	0.541116	0.539866	0.47628	244.6
Construction land	0.000000	0.000000	0.000000	0.000000

**Table 4:** Human activity intensity table.

Parameter	Farmland	Forest	Grass	Lakes and Rivers	Reservoir Pond	Wetlands	Construction Land
Lohani	0.61	0.12	0.61	0.12	0.33	0.38	0.96
Leopold	0.59	0.14	0.59	0.13	0.29	0.42	0.94
Delphi	0.64	0.13	0.64	0.15	0.35	0.55	0.96
Average value	0.61	0.14	0.61	0.13	0.32	0.45	0.95

**Table 5:** Values and changes in ecosystem services in arid and semi-arid ecological regions of China in 2000, 2010, and 2020.

Land-Use Types	2000/USD 100 mn	2010/USD 100 mn	2020/USD 100 mn	2000–2010/%	2010–2020/%	2000–2020/%
Forestland	1996.14	2141.88	2107.83	7.30	−1.59	5.60
Grassland	5223.06	4669.05	4871.16	−10.61	4.33	−6.74
Wetland	851.75	1399.24	1082.36	64.28	−22.65	27.07
Water body	385.34	445.42	680.73	15.59	52.83	76.66
Unused land	247.46	272.38	237.20	10.07	−12.92	−4.15
Cultivated land	564.56	660.55	710.94	17.00	7.63	25.93
Total	9268.30	9588.51	9690.21	3.45	1.06	4.55

**Table 6:** Changes in the structure of ecosystem services between 2000, 2010, and 2020.

Primary Types	Secondary Types	2000/USD100 mn	2010/USD 100 mn	2020/USD 100 mn	2000–2010/%	2010–2020/%	2000–2020/%
Supplying services	Food production	301.00	299.11	312.70	−0.63	4.54	3.89
	Raw material production	414.36	421.03	425.56	1.61	1.08	2.70
Regulating services	Gas regulation	1081.95	1067.72	1080.25	−1.32	1.17	−0.16
	Climate regulation	1307.80	1407.13	1364.41	7.60	−3.04	4.33
	Hydrological regulation	1406.62	1525.48	1569.63	8.45	2.89	11.59
	Waste disposal	1208.62	1340.04	1356.84	10.87	1.25	12.26
Supporting services	Conserve the soil	1457.65	1413.49	1443.11	−3.03	2.10	−1.00
	Maintain biodiversity	1387.72	1383.32	1403.07	−0.32	1.43	1.11
Cultural services	Provide an aesthetic landscape	702.57	731.18	734.64	4.07	0.47	4.57
Total		9268.29	9588.51	9690.22	3.45	1.06	4.55

**Table 7:** Distribution ratio table of human footprint index grades in 2000, 2010, and 2020.

The Human Footprint Index Level	Year 2000	Year 2010	Year 2020
Very low human footprint index	29.96	32.55	25.28
Low human footprint index	23.77	30.20	17.16
Medium human footprint index	45.80	36.40	17.63
High human footprint index	0.41	0.70	19.17
Very high human footprint index	0.06	0.15	20.76

**Table 8:** Ordinary square regression results and spatial regression model test.

R^2^ (OLS)	2000			2005			2010		
	0.190			0.244			0.273		
Test	MI/DF	Value	Prob	MI/DF	Value	Prob	MI/DF	Value	Prob
Moran’s I (error)	0.8510	176.3092	0.000	0.8457	175.2185	0.000	0.7943	164.5630	0.000
LM (lag)	1	29,791.487	0.000	1	28,931.6269	0.000	1	25,829.9915	0.000
Robust LM (lag)	1	289.561	0.000	1	473.8510	0.000	1	671.6147	0.000
LM (error)	1	31,036.977	0.000	1	30,654.6355	0.000	1	27,038.9898	0.000
Robust LM (error)	1	1535.052	0.000	1	2196.8596	0.000	1	1880.6130	0.000
LM (SARMA)	2	31,326.538	0.000	2	31,128.4866	0.000	2	27,710.6045	0.000

**Table 9:** Comparison of the goodness of fit between the spatial error model and the spatial lag model.

	2000		2010		2020	
	SEM	SLM	SEM	SLM	SEM	SLM
R^2^	0.898	0.898	0.901	0.906	0.885	0.886
LogL	−112,851	−112,858	−112,868	−112,909	−113,905	−113,927
AIC	225,708	225,722	225,740	225,823	227,815	227,860
SC	225,723	227,874	225,755	225,845	227,837	227,874

**Table 10:** Regression estimation of the spatial error model.

Variable	2000		2010		2020	
	Coefficient	Standard Deviation	Coefficient	Standard Deviation	Coefficient	Standard Deviation
CONSTANT	0.072	0.0260	0.074	0.008	0.059	0.006
HFI	0.047	0.002	0.080	0.004	0.054	0.003
λλ	0.949	0.003	0.951	0.003	0.944	0.003

*p*  ≤ 0.001.

## Data Availability

Data sharing is not applicable to this article.
